# Employees Perceptions of Job Insecurity and Performance: A Qualitative Approach

**DOI:** 10.3390/ijerph192416665

**Published:** 2022-12-12

**Authors:** Felipe Muñoz Medina, Sergio López Bohle, Sebastian M. Ugarte, Maria José Chambel, Erika Wall

**Affiliations:** 1 Departamento de Tecnologías de Gestión, Facultad Tecnológica, Universidad de Santiago de Chile, Santiago 9170022, Chile; 2 Departamento de Administración, Facultad de Administración y Economía, Universidad de Santiago de Chile, Santiago 9170020, Chile; 3 Faculty of Economics and Business, University of Chile, Santiago 8330015, Chile; 4 Faculdade de Psicologia, Universidade de Lisboa, 1649-013 Lisboa, Portugal; 5 Department of Health Sciences, Mid Sweden University, 831 25 Ostersund, Sweden

**Keywords:** COVID-19, experience, emotions, content analysis, interview study

## Abstract

The purpose of this article is to understand the experience of workers’ perceptions of job insecurity and its relation to performance. To this end, we conducted semi-structured interviews with 38 workers in the retail, services, education, financial, construction, and pharmaceutical industries in Chile. Using content analysis based on workers’ accounts of their own experience, we identified two main categories: (a) the experience of job insecurity viewed in relation to the context of the COVID-19 pandemic and emotional aspects of job insecurity, and (b) the relation between job insecurity and performance. The possibility of job loss expresses itself in experiences and emotions that are related to the performance of workers in different ways. These findings are discussed in terms of stress theory and the motivation to preserve jobs.

## 1. Introduction

The perception of job insecurity has been an issue of growing concern with its increase among workers due to economic, technological, and social changes in recent decades [1,2,3]. More recently, the ongoing COVID-19 pandemic has brought about a global economic crisis, causing restructuring, job suspensions, and massive layoffs, intensifying the feeling of job uncertainty [4,5]. Hence, the perception of job insecurity has become an issue of growing concern in the labor market due to its multiple negative consequences for workers and organizations in general [6,7].

Job insecurity is a phenomenon of perception that can vary from one worker to another, even when faced with the same employment situation [8,9,10]. As job insecurity has usually been defined as the “feeling of powerlessness to maintain the desired continuity in a threatened work situation” [8] (p. 438), it refers to the perceived threats to one’s job as a whole [11,12]. Accordingly, job insecurity is associated with serious negative effects on health and wellbeing. The experiences of job insecurity are related to psychological distress [13], depression [14], and minor psychiatric morbidity [15], as well as to physical outcomes such as heart disease [16], high cholesterol and blood pressure [17], hypertension [18], and obesity [17]. To contribute to the ambitions of the World Health Organization [19] on equality, mental health, and work for all, it is crucial to better understand how job insecurity affects the individual as well as its relation to organizational goals. Hence, the negative outcomes of job insecurity have the potential to negatively affect the organization and its functioning and survival in competitive markets [20].

Performance is one of the most relevant aspects that workers demonstrate in an organizational context [21,22,23], and it has been considered a multidimensional construct [24,25]. It has been defined as actions and behaviors that promote the objectives of the organization [26,27]. Similarly, Rotundo and Sackett [28] define performance as actions and behaviors that are under the control of the individual and contribute to the goals of the organization. Although the consequences of the perception of job insecurity have been extensively investigated, its relation to performance has not yet been fully determined [29,30], posing a considerable challenge in the field [20,31,32].

Quantitative studies have shown a negative link in the relationship between the perception of job insecurity and performance [33,34,35,36] where, for instance, it has been shown in a meta-analysis that the negative effects of job insecurity on performance are more severe among employers with longer tenure than shorter tenure and among older employers than younger [34]. In contrast, other studies have found positive impacts [37,38]. Using a laboratory experiment, Probst has shown that participants faced with the threat of layoff were more productive than others even if at the same time they decreased in creativity [38]. Lastly, other studies have determined that there is no significant link between the variables [12]; hence, there are several plausible factors that may moderate the relationships between job insecurity and its potential outcomes. However, empirical research has neglected the situation of how workers perceive and internalize job insecurity [8,39] and how the experience of job insecurity can be related to performance [20,31,36]. Addressing this gap is the central motivation for our study.

In this regard, understanding how job insecurity is perceived and internalized and how it is related to performance could indicate new avenues for future developments that might be systematically and quantitatively assessed in subsequent studies. Although orientations toward this relationship have been proposed in previous studies, we intend to obtain a better understanding of how workers express this dynamic based on their own experience [40].

Thus, in order to explore the perception of job insecurity of workers and how they view the relation between this job uncertainty and their experience of performance, we frame our study based on fundamental theories explaining the impact of job insecurity on performance, such as stress theory [41] and the motivation for job preservation [32]. From this perspective, we understand performance as a complex phenomenon that takes place in a changing and demanding environment [42,43,44], requiring not only a quantitative approach in order to understand it, but also a qualitative orientation [45,46,47,48] in order to analyze the multiple perspectives of its complexity [49,50,51]. In the same vein, like other research on the performance of workers using a qualitative research design [52,53,54,55], we follow such a research approach in order to explore the underlying aspects [56] of job insecurity and its influence on performance. From this background, the aim of the present study is to analyze how workers experience job insecurity and the relation between job insecurity and performance. Specifically, we focus on the common elements according to the experiences of workers regarding the phenomenon under study [57,58] to address the following questions:

(1) How do workers perceive and internalize the experience of job insecurity?

(2) How does the perception of job insecurity influence the performance of workers?

This study makes important contributions to the literature. First, we study the way in which workers perceive, internalize, and express job insecurity as a relevant experience of their organizational and social context, an aspect on which a consensus has not been reached to date in the literature [8,32,59,60]. Second, we analyze how job insecurity is experienced in relation to worker performance, an issue on which various studies have reported inconsistent results [20,34,59]. Third, while previous studies have been limited to contrasting theoretical views on the impact of job insecurity on job performance [12,34], we adopt a theoretical explanatory approach that presents a more complete vision of how individuals perceive job insecurity and its subsequent influence on performance through stress theories and the motivation to preserve jobs. Fourth, to our knowledge, there are no previous studies applying a qualitative research design to the experience of job insecurity and how it can influence the performance of workers. Moreover, the few qualitative studies specifically analyzing performance have shown results that are different from those of quantitative studies [61]. In this vein, we believe that research in an area mainly addressed through a quantitative research approach needs a qualitative examination so that the experiences of workers are documented and interpreted [62] in order to validate the relationship of the previously investigated phenomena and to deepen understanding on them [63,64,65]. In this way, our research also addresses this gap in the literature [32,34,35,36] by applying a qualitative research design. Finally, from a practical standpoint, our study provides recommendations for decision makers in organizations, who may have information that allows them to understand in greater depth the perception of job insecurity, as well as its influence on the performance of workers.

### Theoretical Framework on Job Insecurity and Performance

Although research has highlighted in recent years various consequences of job insecurity for workers [32,34,60], the analysis of its influence on performance in comparative terms has not received the same interest [36,66,67]. Thus, there are several unanswered questions about how workers experience job insecurity in their organizational context and how job insecurity influences performance [68,69,70].

The literature has proposed various mechanisms to theoretically explain the consequences of job insecurity on the performance of workers. In this research, we used stress theory [41] and the motivation to preserve jobs [32] as our approach. From the point of view of stress theories, job insecurity is a stressor that generates negative consequences for workers and is considered one of the most important stressors in organizations [71]. Stress denotes emotions regarding concerns about the threat or uncertainty of possible job loss [72,73,74]. Job insecurity poses a threat to workers centered on an insufficient ability to collect contextual information on the potential harm of being without a job. In this way, employees express emotional responses according to the stress that this situation has brought about [75]. In the same way, workers seek to obtain, keep, and replace the resources that have been lost [76]. Research has reported a negative relationship between work stress factors and various performance outcomes [35,77]. In this way, workers who are under stressful conditions can limit their efforts toward the goals of the organization in order to keep resources [76], because the effort to perform adequately costs time and energy.

From the perspective of motivation to preserve employment, we understand that job insecurity can motivate workers to carry out behaviors that aim to avoid the possible loss of their job. In this way, workers try to demonstrate their importance to the organization by increasing their performance levels. Fundamentally, the motivation to preserve employment is based on the assumption that performance is crucial to secure the job and counteract the experienced job insecurity. Thus, as job insecurity is a threatening condition to workers, they increase the behaviors that they consider relevant to the organization in order to achieve better assessments from their supervisors than their colleagues [78].

In this way, given the current relevance of job insecurity to workers and organizations in general [5,78], in this study, we focus on the content of the perception of job insecurity associated with its influence on performance from the workers’ perspective in order to increase the understanding of these important dimensions of human resource management, both theoretically and in their practical implications.

## 2. Methods

The purpose of this study is to explore the phenomenon of the perception of job insecurity and its relation to the experienced performance of workers. A qualitative design was used that provides us knowledge about how job insecurity is perceived, and how these experiences of job insecurity are perceived as related to performance. In this way, this qualitative research provides a deeper understanding of workers’ experiences based on a specific context, which allows us to collect information extracted from those same participants [79]. Similarly, analyzing the phenomenon of job insecurity and its relation to performance from a qualitative research perspective will help us to carry out a more complete analysis of what the experience of job insecurity and its perceived influence on workers implies for workers’ performance and will help organize the hitherto conflicting findings in the area [3,35,36,59].

Based on this argument, we use content analysis [56] as a methodological design, allowing us to explore and understand the work experiences of workers and their values, beliefs, and feelings regarding the phenomenon of job insecurity [80,81,82].

### 2.1. Recruitment Procedure and Participants

In accordance with our objective and research questions, a non-random sampling of study participants was used based on the recommendations of Patton [83] and Pietkiewicz and Smith [84]. Participants were recruited among students in four part-time programs of diploma studies in management, human resources, public health, and public policy at a large university in Chile. To be included, each participant must be employed for more than one year in the same company. Hence, dependent/independent contractors were excluded. The sample consisted of 38 participants, who were contacted via email. No incentive was given to participate. All participants were full-time salaried workers from various industries in Chile, including the following: services, education, retail, financial, construction, and pharmaceutical. Women made up 55% of the sample (21 participants). On average, the interviewees had 12 years of work experience and were on average 37 years old. All participants were Chilean nationals.

### 2.2. Interview Guide

Semi-structured interviews were used to collect data, given their potential to provide meanings from the participants themselves while determining relevant emerging issues during the course of the interview [85,86]. In the same way, it allows for the production of possible descriptive and detailed data based on the participants.

The construction of the interview guide was based on previously reviewed literature on job insecurity, performance, stress, and motivation. In turn, the possibility that the participants had the freedom to dialogue about any phenomenon related to those inquired about was allowed for within the conduct of the interviews, on the basis of both their own experiences and personal stories and those of their colleagues at work and in the organization in general.

To meet the objective of this research, we exploited the meaning of the phenomenon of perceived job insecurity within the natural environment of participants. The participants were asked about: (a) their current job and organization, (b) if they had ever experienced a situation of uncertainty about their job continuity, (c) how frequently they had experienced this situation and how long it had lasted, (d) what was experienced and how they could explain that particular experience, and (e) whether and how this experience particularly influenced their job performance.

### 2.3. Data Collection

Data collection was carried out during September and October 2020. All the interviews carried out were recorded through Zoom web platform or Google Meet. Participation in the interviews was voluntary and lasted approximately 35 min each.

To safeguard the ethical aspects of the research, each participant was given an informed consent document explaining the objectives and scope of the study. The information on the study was sent via e-mail as part of the process of recruitment. Additionally, the same written information was given prior to each interview. Likewise, it was indicated that the study was completely confidential and anonymous, and that the information would be used only by the main researcher and for academic purposes. At the same time, each interviewee was assigned a fictitious name and particular code, indicating their gender and a global description of the area or position in which they work, in order to protect their identity. Prior to data collection, the study was examined by the ethical committee of the University of Santiago, Chile, which approved the research regarding ethical considerations.

### 2.4. Data Analysis Procedure

To analyze the data obtained from the semi-structured interviews, content analysis of qualitative data was used following the process described by Graneheim and Lundman [56]. We identified, coded, and created categories based on certain themes or patterns emerging from all material collected. In this way, the data were analyzed based on the theory and epistemological guidelines chosen [87] by means of the central assumption that workers share common situations and problems (perception of job insecurity), from which we generated emerging categories around the main backgrounds and characteristics of job insecurity. Similarly, the link between job insecurity and worker performance was given consideration.

In order to provide internal validity to the results, first, all the semi-structured interviews were transcribed verbatim and read by the main researcher to arrive at a global impression of the data. After this first reading, descriptive and analytical notes were made for each of the interviews, identifying the first content units. The texts were then coded and categorized using the ATLAS.ti 8.0 software. Subsequently, interpretations of the information underlying the story were generated, generating emerging categories based on the experiences of the interviewees and their link with the phenomenon being studied [56], that is, job insecurity and performance. Finally, in a comparative and constructivist way, we carried out a review of the meanings and representations that mediate the contents of the interviews and the interpretation made. All authors agree on the final solution of the analysis. These themes, followed by sub-themes, are presented below in the results section.

## 3. Results

The purpose of this research was to understand the experience of workers’ perception of job insecurity and its relation to their performance. Our results presented below account for the elements that characterize the phenomenon of job insecurity, as well as its experienced relation to performance. These results are developed through an analysis of the experiences and perceptions of the participants. Two main categories were identified: (a) the experience of job insecurity, viewed in relation to the context of the COVID-19 pandemic and the emotional aspects of job insecurity, and (b) the relation between job insecurity and performance, where performance was experienced as increased, decreased, or not changed due to job insecurity.

### 3.1. The Experience of Job Insecurity

From the analysis, we observe that the perception of insecurity in the face of the possibility of losing a job was a highly central experience for the participants. In the material, expressions of job insecurity were clear. It can be exemplified as follows:

*In my case, there is uncertainty and there is also instability because there is no security (…), or simply the feeling of frustration (…) and you have to keep looking for another job. *

*(E33, man, construction site manager)*

*I think that personally I have felt that pressure, that uncertainty of knowing whether I will be without work tomorrow or the day after. *

*(E12, man, education)*

Regarding job insecurity, two sub themes were created based on the material. The first sub-theme reveals how job insecurity was framed by the context of the ongoing pandemic, and the second sub-theme shows how job insecurity was expressed in relation to various emotional aspects.

#### 3.1.1. Contextual Aspects of Job Insecurity

Regarding the perception of uncertainty in the face of a potential job loss by the participants, we note that the COVID-19 pandemic has been a contextual antecedent in our results. In the material, the understanding of the COVID-19 pandemic crisis as a contextual external antecedent of the perception of a potential job loss and of the stress of these experiences was clear, as shown by some participants:

*I believe that the fear of our times, [the fear] of losing one’s job, has currently had greater emphasis with the health crisis that we are experiencing. I believe that this unfavorable panorama that we have to go through leads one to have this constant thought of the possibility of losing your job, burnout, demotivation, the anxiety involved, and I think it is in the times in which we are living that I have felt it the most. I think that, previously, at some point one can be subject to a company-specific situation, but not at the level as we are experiencing it now.*

*(E15, woman, head of salary processing)*

*There is always that fear, especially now with the pandemic, that they want to reduce staff, that they want to reduce our salaries and all that (…), and I believe that, if that were to happen this year (…) there would be super big uncertainty because the bank is not reaching the figures obviously because of all that is happening. *

*(E6, female, human resources psychologist)*

The contextual perspective on job insecurity pinpoints the consequences of the pandemic in terms of a deteriorating economy but also how the context of the pandemic is viewed in terms of a general crisis affecting work as well as private life, as illuminated in the second quotation (above).

#### 3.1.2. Emotional Aspects of Job Insecurity

The interpretation of the experience of job insecurity found in the material showed that job insecurity was not only viewed in relation to the context of the pandemic. Even more central in the material was how job insecurity was viewed as associated with the emotions triggered by a potential threat to one’s job. The most central emotional expressions found in the analysis are uncertainty, anxiety, stress, frustration, and fear. Below are some accounts by the interviewees that give an account of the emotions expressed:

*You have something for sure, I don’t know, for five, six, ten years and suddenly things that you have never seen begin to happen and that kind of uncertainty is generated, of what is going to happen, that fear, fear of the unknown, I think. And that fear of the unknown is what generates all these feelings such as anxiety or the cause of frustration, of not being able to do something, then in the end when they take you out of your comfort zone it is what generates this fear, this uncertainty. *

*(E22, male, financial analyst)*

*A certain uncertainty that you do not know when, what will happen in the short term, and the rest, perhaps it gives you a bit of, I don’t know if it is frustration, but yes, it could be a bit of frustration, like a process that one says heck, a while ago I had everything ready and from one moment to the next it is gone. *

*(E5, man, education)*

The analysis showed that the emotions expressed as associated with the experiences of job insecurity are associated with the feeling of seeing a blockage of one’s options to control, in some sense, the perceived work situation.

### 3.2. The Relation between Job Insecurity and Performance

Regarding the possible relation between job insecurity and performance, the analysis revealed diverse responses, here presented as three parallel sub-themes. The most central in the material was that job uncertainty was viewed as having a negative impacts on performance. Another perspective on the relationship between job insecurity and performance found in the material was that job insecurity has a positive bearing on experienced performance. Lastly, the analysis revealed a third perspective, namely that the feeling of job uncertainty is not experienced as relevant regarding performance.

#### 3.2.1. Job Insecurity Is Experienced as Reducing Performance

In conditions of uncertainty about their future employment, the most common perspective in the material analyzed is that performance is experienced as diminished, as seen in the following quotations:

*I think there is here an impact [on performance], yes, I noticed it to be honest, I’m not going to lie to you, but there is indeed an impact, but it hasn’t been that big, if you ask me, a marginal impact. But it does show, I [sic] all the commitments that I have clear, the most important, most urgent things, we do it immediately and we postpone the others. *

*(E5, man, education)*

*I am going to give an example, of writing an article where one must be very concentrated, read literature, then establish a solid argument to write and your mind is halfway through thinking (…) am I going to proceed today? Is this going to be worthwhile? Because one of these days I am left with nothing, then of course, yes, anyway I think it affects you, even if one tries to take it in the best way, it affects you, I could not say otherwise. *

*(E19, man, education)*

The main explanation found in the analysis for the experienced decrease in performance due to job insecurity is that they would allocate resources (such as attention or time) to concerns about a potential job exit, and those resources are therefore drawn away from the work function.

#### 3.2.2. Job Insecurity Is Experienced as Increasing Performance

The results of the analysis indicate that parallel to the experiences of decreased performance, presented above, another perspective evident in the material is the experiences of where participants viewed greater energy gained due to the perception of job insecurity. Such perspectives are exemplified in the following quotations:

*In my experience, it affects me in a positive way. In the sense of working, that, feeling this kind of uncertainty, makes one to be more, perhaps more constant, more studious, or strict with schedules, with the way of working, with the adherence [to work], deep down with trying to get things done faster. *

*(E7, man, lawyer)*

*They can change your boss, your deputy boss, and they can change the team, and if you don’t show that you are indeed productive, you’re efficient, you produce good work, etc. (…) then I think that at that minute it scared me and obviously I wanted to do it well and make as few errors as possible (…) [job insecurity] makes a greater case for my way of working, never feeling safe and always doing it well. *

*(E8, female, human resources manager)*

As illuminated in the quotations, the analysis showed that the participants made efforts to demonstrate the expected results within the deadlines agreed upon by the organization, even hastening work processes. This strategy translates into increased performance levels as protection against the latent threat of losing the job.

#### 3.2.3. Job Insecurity Is Experienced as Not Related to Performance

Lastly, the experience of not perceiving a relation between performance and the potential threat of job loss was found in the analysis. This can be evidenced in the following accounts:

*In terms of performance, I am quite neutral, that is, I do not perform well or badly, in the end I do my job. *

*(E38, woman, sales)*

*I try not to be affected by external situations [organizational events] in my performance. I try not to be affected in my daily work by something that may be happening outside (…) no, I would not react. *

*(E12, male, university academic)*

As the quotations illuminate, performance is here expressed as related to the work itself, rather than understood in relation to the circumstances, such as the occurrence of job insecurity.

## 4. Discussion

The objective of this study was to understand the experience of workers’ perception of job insecurity and how it can be related to their performance. As illuminated, it is known that job insecurity has negative effects on individual health [15] and is also related to the possibilities for the organizations reaching goals and succeeding in the global market [20]. From this, in-depth knowledge on the relation between job insecurity and performance is important on an individual as well as an organizational level. Such knowledge can contribute to the goals of health and work for all, as stated by the World Health Organization [19]. Here, the results of the analysis are discussed in relation to the theoretical framework and previous research.

### 4.1. The Experience of Job Insecurity

Regarding job insecurity, the analysis revealed how job insecurity was viewed from a contextual perspective and an emotional perspective. From a contextual perspective, the analysis reveals that job insecurity was viewed in relation to the economic consequences of the pandemic [7,88], such as the stagnation of new projects, work stoppages, and different measures, which have prevented the development of employers [7]. From this perspective and using the theory of scarcity (Mani et al., 2013), workers are in a position where various factors affect them, with the COVID-19 pandemic being a highly relevant element. Workers report possessing fewer resources to cope with the many demands of everyday life, where the perceived shortages [89] of such items as disinfection supplies, health, family support, food, and jobs divert the cognitive resources and professional capacities of individuals, thus increasing their perception of job insecurity. The main factor responsible for this imbalance would be the current COVID-19 pandemic. As other studies have reported [90], the interviewees express not only concern but alarm due to the economic context of the COVID-19 pandemic. To this, they also add concerns specific to everyday life, which has been changed because of social distancing policies, quarantines, prohibition of non-core activities, and the fear of the consequences of getting infected [91].

Despite the general influence of the COVID-19 pandemic on job insecurity, job insecurity as an experience is an individual event. Even when workers have the same working conditions, their experiences of uncertainty about the future of work may be different [36]. Here, such variations were found in how job insecurity was viewed from an emotional perspective revealing a wide range of emotions. However, most of the experiences that the analysis uncovered are related to emotions of uncertainty, anxiety, stress, and frustration. This is consistent with previous definitions by different authors of job insecurity [12,73]. These emotions denote the concerns of the threat of uncertainty about possible job loss [73,74], as well as stress caused by the experience [72]. In turn, frustration is evidenced by previous literature as a sensation experienced due to job insecurity, where workers who want stability experience frustration due to the threat of uncertain work conditions [12,92]. Employees see even their basic needs frustrated due to the perception of job insecurity [74,93]. In this way, our findings report the expression of various emotions by workers, where most emotional responses, but not all, have as a common element the fact that they consider job insecurity as a stressor. The emotions and experiences revealed by the analysis are consistent with previous research findings showing the perception of job insecurity as a stressful condition in itself [73,94]. From this perspective, stress theory explains that stressors such as job insecurity are associated with negative emotions [77] or expressed through negative attitudes or behaviors [41].

### 4.2. The Relation between Job Insecurity and Performance

When it comes to the second theme of the analysis, the relation between job insecurity and performance, the analysis gave a diverse picture. Most central was the experienced negative effect of job insecurity on performance, but at the same time the material included perspectives on increased performance as well as the expressions of no experienced relationship between job insecurity and performance.

The first sub-theme where performance is viewed as negatively affected by job insecurity is consistent with previous research that indicates a negative impact of job insecurity on performance [34,36,95,96]. This link can be explained through stress theory, which explains that since job insecurity is a stressor for workers, they cognitively perceive, process, and interpret the stress situation [41], resulting in a reduction in their attention that decreases performance [97]. This event, in which job insecurity influences the performance of workers, is evidenced in most of the reports in our study, constituting a challenge and a constant emotional burden for workers. The positive experiences of performance due to job insecurity, presented in the second sub-theme in this part of the analysis, are also consistent with previous studies [3] that found that the feeling of job uncertainty can drive workers to take actions to avoid job loss. In this vein, we explain the results of present study from the perspective of job preservation [32,78], where workers focus on actions that allow them to preserve their jobs. Along these lines, they increase their efforts in the organization in order to be noticed and be considered valuable workers [32,98]. In this way, they hope that a decision for dismissal will not be made after their efforts to preserve their job [3,97] by differentiating themselves positively from other workers exposed to the same downsizing threat. In relation to the various results found in previous studies, the third perspective of the analysis, where no relationship between job insecurity and performance were expressed in the material, is also supported by previous research [12,99]. A possible and very simple explanation is that job insecurity as an experience simply does not influence the performance of workers. On the other hand, it could happen that the influence of job insecurity is determined according to certain contexts and particular types of performance [12]. Another explanation may be due to the fact that the workers interviewed may show a limited perception of job insecurity, given that, despite the circumstances, they continuously demonstrate good performance. In this way, it is possible that they did not feel the influence that the uncertainty of a possible dismissal had on their performance levels.

The evidence in this latter section of the analysis mainly presents negative consequences of job insecurity on performance, as indicated in the literature [34]. However, as previously mentioned, the link between job insecurity and performance has been less addressed in research and its consequences are still not entirely conclusive [21,59]. Although there is much evidence in the quantitative literature of the negative impact of job insecurity on performance [35,36], positive consequences have also been reported [3,37]. This variability in effects is evidenced in the results of the analysis in the present study. However, even beyond the number of respondents reporting a given kind of influence, we believe that our findings provide basic guidance in focusing on the existence of different interpretations on the relation between job insecurity and performance.

## 5. Conclusions

Our results indicate that the interviewees in the study have perceived job insecurity in their career, particularly during the COVID-19 pandemic. In turn, our findings are generally consistent with previous research and show that: a) job insecurity includes the background of external organizational and contextual events; b) job insecurity is experienced through emotions that describe elements of stress; and c) job insecurity has three possible consequences for performance—it can decrease or increase job performance, or else not have a clear connection to performance.

We note that workers frequently experience job insecurity. However, it can be an experience with multiple negative consequences for workers as well as for organizations. In this vein, we confirm some of the theoretical assumptions of previous research, and through a qualitative study, we provide new information when interpreting each of these findings.

First, the way in which workers perceive, internalize, and express job insecurity is relevant because it reflects not only the organizational context, but also exogenous events such as the COVID-19 pandemic that are not under the control of organizations or the individual. In this sense, the fact that there is no commonly accepted definition of job insecurity and what it implies for workers [32,36] indicates that the backgrounds of the perception of job uncertainty are multiple and diverse.

Second, we analyze how job insecurity is related to worker performance. In this way, based on our findings, we corroborate previous research indicating that the impact of job insecurity on performance is not entirely conclusive [32,59]. Based on this vein of argument, the heterogeneous results discussed here and presented by the employees in our sample, where job insecurity can either decrease, increase, or not be experienced as affecting workers’ job performance only confirms that this line of research still needs development [31,66]. Thus, in our study, we seek to extend earlier results by presenting new evidence in this area, as we believe that the interpretations of job insecurity and performance differ across workers. This is reinforced by some studies demonstrating the complexity of the impact of job insecurity on performance [59,100]. In this way, based on our findings and previous research, it is necessary to continue exploring the issue in order to clarify the relation between job insecurity and performance by applying different theoretical perspectives, methodological designs, and more specific performance dimensions.

Third, while previous studies have been limited to the discussion of different theoretical perspectives on the influence of job insecurity on performance, regarding which theory has not yet found clarity [12,34,100], our perspective is the integration and complementarity of two theoretical frameworks capable of explaining the results presented here. Stress theory predicts the negative impact of insecurity on performance, while motivation theory for job preservation explains the positive impact. Thus, we do not intend to favor one theory over the other in terms of explanatory relevance.

Fourth, based on a qualitative research design, we examined the quantitative research in the area and complemented it by interpreting the experiences and emotions of workers regarding job insecurity and its relation to performance. This allows us to address a methodological gap in the literature on this phenomenon.

Fifth, from a practical point of view, decision makers in organizations can encourage their employees to feel confident in expressing their concerns regarding job insecurity in order to establish strategies and procedures that allow (a) a deep understanding of the experience of job insecurity and (b) the manifestations of job insecurity in the worker’s performance, and above all measures to reduce the negative effects that job insecurity could generate. Based on this, the breadth of the findings presented here indicate an orientation for actions to reduce job insecurity due to its harmful effects on the well-being of workers, as well as the development of strategies that allow job insecurity to be controlled with a focus on increased performance.

### Limitations and Future Research

Despite the contributions of this research to our understanding of this subject, we identified some potential limitations that should be mentioned. First, the diversity of labor sectors from which the sample of participants comes might have diminished the depth of experiences of job insecurity in specific work environments reflecting a particular industry or organization. Second, our sample included participants who had formal studies and completed specialization and postgraduate programs. This particular group is likely to be less affected by perceptions of job insecurity than other groups with a lower level of formal education [94,101]. Third, the number of participants in this study and the inherent nature of the qualitative approach that we have used (semi-structured interviews) do not allow a generalization of the results presented. From the perspective of future research, new studies in the area could address further the experience of job insecurity and its relation to and possible influence on performance in industry-specific contexts to further explore how contextual factors can deepen the understanding of these issues. Further, the gender and intersectional perspectives on the conditions of working life should be included in upcoming studies. Additionally, we argue that further research should focus on determining specific dimensions of performance understood by employees—for example, contextual performance or innovation performance. In the same way, the COVID-19 pandemic has profoundly modified social and organizational environments worldwide, reducing the productive activity of companies and causing the suspension of labor contracts or massive layoffs. Along these lines, future research should also address the post-pandemic effects on job insecurity and worker performance.

## Data Availability

The data that support the findings of this study are available from the corresponding author upon reasonable request.
